# The integrin adhesome network at a glance

**DOI:** 10.1242/jcs.192054

**Published:** 2016-11-15

**Authors:** Edward R. Horton, Jonathan D. Humphries, Jenny James, Matthew C. Jones, Janet A. Askari, Martin J. Humphries

**Affiliations:** Wellcome Trust Centre for Cell-Matrix Research, Faculty of Biology, Medicine and Health, University of Manchester, Manchester M13 9PT, UK

**Keywords:** Adhesion, Cytoskeleton, Integrin, Integrin adhesion complex, Adhesome

## Abstract

The adhesion nexus is the site at which integrin receptors bridge intracellular cytoskeletal and extracellular matrix networks. The connection between integrins and the cytoskeleton is mediated by a dynamic integrin adhesion complex (IAC), the components of which transduce chemical and mechanical signals to control a multitude of cellular functions. In this Cell Science at a Glance article and the accompanying poster, we integrate the consensus adhesome, a set of 60 proteins that have been most commonly identified in isolated IAC proteomes, with the literature-curated adhesome, a theoretical network that has been assembled through scholarly analysis of proteins that localise to IACs. The resulting IAC network, which comprises four broad signalling and actin-bridging axes, provides a platform for future studies of the regulation and function of the adhesion nexus in health and disease.

## Abstract (precis)

**Summary:** We have generated a new depiction of the integrin adhesome network that integrates experimentally derived IAC proteomes with the literature-curated adhesome to bridge the knowledge gap between these two resources.

## Introduction

In a classic series of resource articles and reviews published between 2001 and 2014, Geiger and colleagues defined, and then refined, the ‘literature-curated integrin adhesome’ (Geiger and Yamada, 2011; Winograd-Katz et al., 2014; Zaidel-Bar and Geiger, 2010; Zaidel-Bar et al., 2007; Zamir and Geiger, 2001a,b). This theoretical network of more than 200 components, constructed *in silico*, contains all the proteins that have been reported to locate to, or regulate, the adhesion nexus (Winograd-Katz et al., 2014). The generation of the literature-curated adhesome has transformed thinking in the field and greatly facilitated candidate-based approaches to address questions such as how signals are transduced across the network, how these signals are influenced by the extracellular and intracellular environments, and how the complex assembles and disassembles. Despite its undoubted value, the network that connects the adhesome components is nonetheless theoretical and a long-standing question has been how it relates to the actual composition of integrin adhesion complexes (IACs). To address this issue, several laboratories have recently developed methodologies to isolate IACs, and used mass-spectrometry-based proteomics to determine their composition (Geiger and Zaidel-Bar, 2012).

**Figure:**
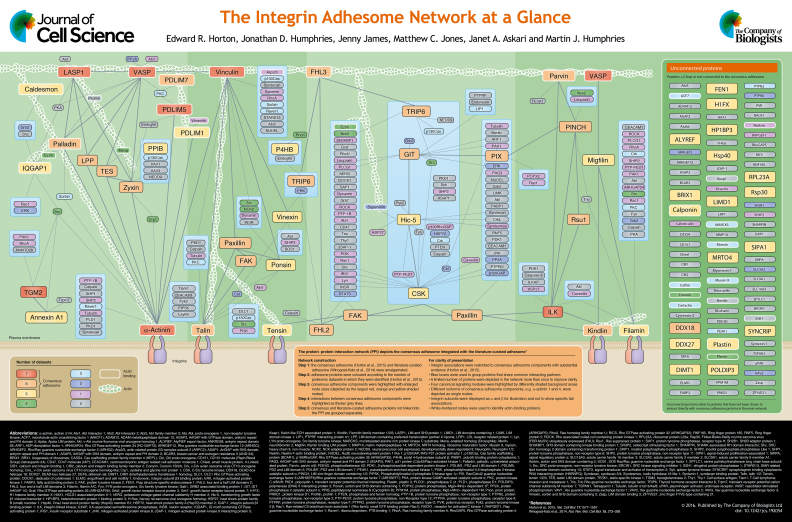

Initial candidate-based proteomic studies of integrins and IAC components identified post-translational modifications and/or interacting partners (Humphries et al., 2015); however, a step change came with the development of methods to isolate integrin-containing ventral membrane preparations. These methods relied on stabilisation of IACs using chemical cross-linkers and enrichment of IAC components by removal of the cell body and cytoplasmic proteins (Jones et al., 2015; Kuo et al., 2012). Mass spectrometry was then used to determine IAC compositions for several cell types under a variety of culture conditions (Ajeian et al., 2016; Byron et al., 2012, 2015; Horton et al., 2015, 2016a; Huang et al., 2014; Humphries et al., 2009; Kuo et al., 2011; Ng et al., 2014; Robertson et al., 2015; Salmela et al., 2016; Schiller et al., 2011, 2013; Yue et al., 2014). Seven of these mass spectrometry datasets, which were generated from different laboratories using diverse methods and from multiple cell types, were used to create a ‘meta-adhesome’ database of more than 2000 proteins that were enriched at fibronectin-induced IACs (Horton et al., 2015). Although the large increase in scale over the literature-curated adhesome might be caused in part by the non-specific isolation of a number of components, it is likely to be a true reflection of the complexity of IAC preparations (Geiger and Zaidel-Bar, 2012). The adhesion nexus is a membrane–cytoskeleton junction, and not a discrete organelle, and therefore the preparations are in effect specific membrane patches proximal to the sites of extracellular matrix (ECM)–cytoskeleton interaction. As the role of these junctions is to control signalling in a spatial manner, the identity of the proteins that are attracted to these sites is likely to be highly relevant. In this Cell Science at a Glance article, we describe the generation of a new depiction of the integrin adhesome network (see poster) in which we have assembled an integrated network of the experimentally derived IAC proteomes and the literature-curated adhesome components, and their interactions, to bridge the knowledge gap between these two resources.

## An integrated view of the experimental and literature-curated adhesomes

Mass-spectrometry-based proteomics provides both a contrasting and complementary view to that provided by the literature-curated adhesome. An emergent property of the meta-adhesome described above is the definition of an IAC core of 60 proteins, termed the ‘consensus adhesome’ (Horton et al., 2015), which represents the most frequently identified components in IAC proteomes (operationally defined as those proteins observed in five or more of the seven datasets). Thirty-one of these proteins were present in the literature-curated adhesome, which indicates that a number of commonly isolated components are either under-appreciated or non-specific. One prominent IAC dataset (Kuo et al., 2011) was not included in the meta-adhesome analysis due to the lack of a negative control ligand condition, but retrospective analysis of this dataset identified a comparable number of consensus adhesome components to the controlled datasets (42/60). This finding both validated the selection of consensus adhesome proteins and demonstrated its value as a resource to streamline analysis of other datasets.

Here, we present a new and updated view of the consensus adhesome that has been integrated with the literature-curated adhesome (see poster). As the consensus adhesome likely represents the core structural components of IACs (i.e. those that are more abundant and stable, and therefore more likely to be retained in ventral membrane preparations), this perspective provides insights into how other less-abundant IAC components present in the literature-curated adhesome might link into this core complex. To construct this integrated network, proteins from the consensus adhesome (Horton et al., 2015) and literature-curated adhesome (Winograd-Katz et al., 2014) were amalgamated and arranged to highlight the consensus adhesome proteins (depicted as the largest nodes shaded red, orange and yellow in the poster, with inter-adhesome interactions represented by thicker grey lines) and their immediate interacting partners. All literature-curated components are included, even if they have not been detected in the isolated IAC preparations. The less-abundant components are coloured green, blue or (for those not detected) grey. This results in the construction of a network comprising the consensus adhesome plus its ‘1-hop’ neighbourhood in the context of protein–protein interactions (PPIs) from the literature-curated adhesome (Winograd-Katz et al., 2014; Zaidel-Bar et al., 2007) and a global database of PPIs (Byron et al., 2015; Chautard et al., 2009; Wu et al., 2009).

To simplify this new IAC landscape, (1) proteins that have been reported to bind actin are shown with white borders; (2) because integrin associations have been reviewed elsewhere (Bouvard et al., 2013; Morse et al., 2014; Iwamoto and Calderwood, 2015), they are restricted here to show only consensus adhesome components with substantial evidence (Horton et al., 2015); (3) proteins that share common interacting partners are grouped within boxes; (4) some proteins are depicted in the network more than once to enable all reported interactions to be visualised clearly; and (5) proteins not incorporated by this strategy are displayed separately (see poster). Some proteins that were unconnected to the main IAC network in the context of the consensus adhesome alone have been reported to bind other known IAC components reported in the literature-curated adhesome, and are therefore drawn into the new network. For example, PDZ and LIM domain 5 (PDLIM5) and PDZ and LIM domain 7 (PDLIM7) link to vasodilator-stimulated phosphoprotein (VASP) through PKC, and IQ motif containing GTPase-activating protein 1 (IQGAP1) links to the network through interactions with Src and ezrin. In addition, visual inspection of the network demonstrates that the consensus adhesome proteins that recruit the highest number of non-consensus adhesome proteins to IACs are filamin, paxillin, focal adhesion kinase (FAK, also known as PTK2), talin, vinculin and α-actinin, suggesting that these proteins might form links to, or regulate, the recruitment of other associated adhesome components.

When the literature-curated adhesome is compared to the consensus adhesome, it is apparent that the consensus adhesome is largely dominated by intrinsic, structural IAC components, such as actin regulators and adaptor proteins, rather than associated proteins, such as guanine nucleotide exchange factors, GTPase-activating proteins and kinases. However, not all actin-binding proteins were identified by these analyses, suggesting that those present represent a specific subset of actin-binding proteins that localise at the ends of actin fibres that are proximal to IACs. Adaptor proteins, such as FAK and paxillin, were identified, whereas a large number of binding partners for these proteins in the literature-curated adhesome were not seen in the consensus adhesome. This might reflect the transient signalling interactions that occur in the highly dynamic environment of the adhesion nexus. In this regard, fluorescence recovery after photobleaching (FRAP) studies have shown large variations in the dynamic exchange of different adhesome proteins within adhesive structures (Carisey et al., 2013; Hoffmann et al., 2014; Kanchanawong et al., 2010; Lavelin et al., 2013; Lele et al., 2008; Rossier et al., 2012; Shtengel et al., 2009; Wolfenson et al., 2013). Nevertheless, consensus adhesome proteins span the full range of these reported dynamics, and therefore the consensus adhesome does not simply represent more stable interactions. Rather, it appears to be the nature of the interaction rather than the dynamic stability of a protein within the complex that dictates whether it was captured by IAC isolation and proteomic strategies.

Analysis of consensus adhesome PPIs suggests that the core adhesome machinery forms the structural connection between integrins and actin, and that this link can be broadly divided into four canonical signalling modules (Horton et al., 2015), as highlighted by the differently shaded green background areas on the poster. These modules include one that contains α-actinin and zyxin family members, a second of vinculin, talin and the vinculin-binding proteins vinexin and ponsin, a third, which contains FAK and paxillin, and a final one made up of two sub-modules that are connected through a kindlin–integrin-linked kinase (ILK) interaction. These four modules were identified based on known signalling axes and integrin–actin links reported in the literature, and are generally supportive of the so-called vertical *z*-plane model in which proteins were observed to occupy specific strata within IACs (Kanchanawong et al., 2010). Viewing the integrated consensus and literature-curated adhesomes in relation to these axes allows visualisation of routes that connect integrins to actin, thereby linking these interactions with known adhesion signalling pathways (see poster). The flattened 2D depiction of a structure such as an IAC that is most likely organised and occupies a complex and interwoven 3D topography can result in a separation of certain proteins and interactions within the displayed network. For example, the separation of kindlin- and filamin-associated proteins has been exacerbated in the network due to the large number of FAK- and paxillin-binding proteins. The four modules are, however, interconnected, and further work is required to test just how discrete they are.

A limitation of the network reported here is of course the absence of interactions that have yet to be identified! For example, there might be proteins in the consensus and literature-curated adhesomes that have key connecting roles, but are not indicated by this network. Typically, poorly studied proteins have fewer reported interactions, and highly connected proteins within the interactome represent well-studied proteins (Rolland et al., 2014). For example, the most connected proteins within the consensus adhesome are well-studied literature-curated adhesome proteins (e.g. FAK, 15 interactions; β1 integrin, 13 interactions; paxillin, 12 interactions). In addition, false-positive interactions within these networks are often present, as *in vitro* assays used to detect PPIs can report interactions that do not occur *in vivo*. Although all of these possible interactions within a network are shown, the protein–protein map is not static, with some interactions only occurring under certain conditions and in certain cell types (Köster et al., 2012). Furthermore, the interactome used to construct the consensus adhesome does not depict directionality within the network (i.e. activating or inhibiting relationships are not shown) and does not take into account the localisation of the reported interactions in cells. These issues will resolve over time as a fuller picture of the PPI landscape evolves and current PPI databases become better annotated. However, these limitations should be considered when interpreting data and seeking biological insights into the signalling mechanisms at IACs as indicated by PPI networks. For this reason, experimental evidence for important interactions needs to be carefully reviewed, and any low confidence interactions should be experimentally validated before drawing any conclusions.

Finally, in the current view of the IAC network, some proteins, including 18 consensus adhesome components, could not be incorporated due to the lack of evidence for their interaction with other components (shown in a separate box on the poster). On the one hand, this might suggest that these components could be non-specific, co-purifying contaminants, despite the use of stringent controlled datasets. However, these proteins might equally be new, understudied adhesion proteins that have not yet been characterised sufficiently to provide PPI data to link them into the network. For example, immunofluorescence analysis has confirmed the localisation of two unexpected consensus adhesome proteins, Rsu1 and caldesmon, to IACs (Horton et al., 2015), suggesting they do play a role in the adhesion machinery. This provides confidence that other unexpected components might also be relevant to adhesion structures or signalling (Byron and Frame, 2016). In addition, previously unidentified actin-binding proteins have been isolated with known adhesion-related proteins. It is therefore likely that new actin linkers are involved in adhesion function and might act to stabilise the integrin–actin link, for example, PDLIM1 and PDLIM5, by bridging the connection between integrin, α-actinin and actin.

## The consensus adhesome and human disease

The association between the literature-curated adhesome components and human disease has been extensively reviewed (Winograd-Katz et al., 2014). Similarly, the relationship of the consensus adhesome proteins to human disorders has been determined by reference to the online Mendelian inheritance in man (OMIM) database (http://www.omim.org/), and it was found that mutations in nine of these genes cause diseases involving the renal, haematological, cardiac, skeletal and muscular systems, as well as certain types of cancer (Horton et al., 2015). In addition, further interrogation of the OMIM indicated that a mutation of a tenth consensus adhesome protein, prolyl 4-hydroxylase subunit beta (P4HB), is implicated in Cole–Carpenter syndrome, a rare bone disease (Cole and Carpenter, 1987; Rauch et al., 2015). This underscores the importance of adhesion signalling to human health given that perturbations impact on several major organ systems.

To complement these analyses, we have further analysed the role of the consensus adhesome proteins using Ingenuity Pathway Analysis (IPA; http://www.ingenuity.com/), which uncovered the potential involvement of a larger number of these molecules in a variety of diseases not revealed by single gene interrogation (Table S1). According to the Disease, Injury and Function output of IPA, the largest disease association was with cancer with over 50% of the consensus adhesome genes (32 out of 60) implicated in breast and/or colorectal cancer alone. Other diseases predicted by IPA to involve the consensus adhesome proteins include the cardiovascular, renal, haematological and musculoskeletal systems already catalogued by OMIM analysis (Horton et al., 2015). IPA analysis also indicated the potential involvement of 16 consensus adhesome proteins in viral infectious disease, which included HIV-1 and West Nile virus. Furthermore, 17 consensus adhesome proteins are predicted to contribute to the inflammatory response and immune cell trafficking, both of which are important processes in the pathology of autoimmune diseases and infection. Taken together, these analyses indicate that almost all of the 60 consensus adhesome proteins play, either singularly or collectively, important roles in human health and disease. Such analysis is therefore useful to inform new avenues for further research into the association of the consensus adhesome proteins with human disease, potentially leading to novel therapeutic targets.

## Summary and future perspectives

Here, we relate the consensus adhesome (Horton et al., 2015), which comprises the most commonly identified proteins in fibronectin-induced proteomic IAC datasets, to the literature-curated adhesome (Winograd-Katz et al., 2014) that was assembled as a theoretical concept from all the proteins that have been reported to locate to, or regulate, IACs. This fusion highlights connections within and between consensus and literature-curated adhesome components and is intended to provide clues to regulatory mechanisms that might control IAC formation, disassembly and signal transduction.

It should be noted that adhesome networks are not fixed, but will continue to evolve with further experimentation. The use of additional cell types and ECM ligands will lead to network refinement and differentiation based on different experimental conditions and proximity-based proteomic techniques (such as BioID and APEX; Rees et al., 2015; Roux et al., 2012) have the potential to reveal additional insights into the composition, and topography, of IACs and adhesion signalling (Dong et al., 2016; Guo et al., 2014). As an example, proximity labelling of paxillin and kindlin recently identified proteins that are consistent with many aspects of the consensus network, but suggested several previously unknown IAC components (Dong et al., 2016). These new components included the adaptor protein KN motif and ankyrin repeat domains 2 (Kank2) that could form a connection between the paxillin and kindlin arms of the consensus signalling axes (Bledzka et al., 2016; Dong et al., 2016; Theodosiou et al., 2016). Interestingly, Kank2 was detected in many proteomic IAC datasets, and only just failed to be included in the consensus adhesome list as it was observed in only four of the seven datasets used (Horton et al., 2015). This further highlights the fact that many meta-adhesome proteins might play important adhesion regulatory or signalling roles. For example, among the proteins that are represented in three or four of the seven datasets that have been used to create the meta-adhesome are Src, breast cancer anti-estrogen resistance 1 (p130Cas, also known as BCAR1), ezrin, NCK adaptor protein 2 (Nck2) and protein enabled homologue (Mena, also known as ENAH).

An additional point to consider is that the IAC isolation procedure does not distinguish between different types of IAC structures and therefore does not account for heterogeneity (Humphries et al., 2015). For example, proteins that might be less abundant in nascent adhesions might not be consistently identified by mass spectrometry. Therefore, integration of the consensus and literature-curated adhesomes might provide useful insights into mechanisms that regulate IACs and indicate how different structural arms of the consensus adhesome might be connected. Furthermore, the consensus adhesome was generated primarily from the early phase of cells spreading on fibronectin, and therefore its composition reflects components that are recruited to α5β1 and αVβ3 integrins at relatively early time points. In contrast, the literature-curated adhesome does not distinguish between ligand or integrin heterodimer, and, therefore, a future comparison with IACs enriched from other conditions, such as other cell types (e.g. epithelial cells), cells spread on different ECM ligands, cells left in long-term culture or IACs isolated from an *in vivo* setting would be instructive. Additional analyses such as these might reveal not only context-specific adhesome components but also the number of consensus adhesome components that are conserved between integrin adhesions in different systems. The role of the consensus adhesome in relation to mechanosensation was recently reviewed, and its components were shown to change almost uniformly upon loss of myosin-driven intracellular force (Horton et al., 2016b). Thus, data generated under alternative conditions, such as using differing ECMs, might present a completely different view of the adhesion nexus or consensus adhesome, or increase the number of literature-curated adhesome components identified using mass-spectrometry-based approaches. In this regard, the available evidence suggests that the consensus composition is determined by the integrin-ligand combination, as only a restricted set of consensus adhesome proteins (10 out of 60) were identified in IAC proteomic datasets generated using the canonical α4β1 ligand, vascular cell adhesion molecule-1 (VCAM-1; Humphries et al., 2009; Byron et al., 2012; Horton et al., 2016b).

Several key questions remain, such as how the composition and organisation of IACs differ from 2D cultures to 3D culture or *in vivo*, and whether a systems-based view of signal relay mechanisms that occur across the adhesion nexus can be obtained. For the former, proximity-based labelling approaches appear to offer the clearest way forward to defining IACs *in vivo*, as isolation strategies for IACs from tissues have not been established. Furthermore, in an effort to obtain a systems view of signalling, a recent proteomic study has shed light on the nature of FAK and Src signalling at IACs, illustrating how IAC composition and phosphotyrosine-based signalling could be independently regulated to control migration and proliferation through alterations in the dynamic exchange of signalling proteins (Horton et al., 2016a). In summary, the consensus and less refined meta-adhesome offer intriguing insights into IAC structure and function, and future experiments, including those investigating the roles of their components in adhesion signalling (Alanko et al., 2015; Robertson et al., 2015; Sarhan et al., 2016), mechanotransduction (Horton et al., 2016b; Schiller and Fässler, 2013) and disease, will no doubt inform their further development and use as a resource for the community.

## References

[JCS192054C1] Ajeian J. N., Horton E. R., Astudillo P., Byron A., Askari J. A., Millon-Frémillon A., Knight D., Kimber S. J., Humphries M. J. and Humphries J. D. (2016). Proteomic analysis of integrin-associated complexes from mesenchymal stem cells. *Proteomics Clin. Appl.* 10, 51-57. 10.1002/prca.201500033 26147903 PMC4737105

[JCS192054C2] Alanko J., Mai A., Jacquemet G., Schauer K., Kaukonen R., Saari M., Goud B. and Ivaska J. (2015). Integrin endosomal signalling suppresses anoikis. *Nat. Cell Biol.* 17, 1412-1421. 10.1038/ncb3250 26436690 PMC4890650

[JCS192054C3] Bledzka K., Bialkowska K., Sossey-Alaoui K., Vaynberg J., Pluskota E., Qin J. and Plow E. F. (2016). Kindlin-2 directly binds actin and regulates integrin outside-in signaling. *J. Cell Biol.* 213, 97-108. 10.1083/jcb.201501006 27044892 PMC4828686

[JCS192054C4] Bouvard D., Pouwels J., De Franceschi N. and Ivaska J. (2013). Integrin inactivators: balancing cellular functions in vitro and in vivo. *Nat. Rev. Mol. Cell Biol.* 14, 432-444. 10.1038/nrm3599 23719537

[JCS192054C5] Byron A. and Frame M. C. (2016). Adhesion protein networks reveal functions proximal and distal to cell-matrix contacts. *Curr. Opin. Cell Biol.* 39, 93-100. 10.1016/j.ceb.2016.02.013 26930633 PMC5094910

[JCS192054C6] Byron A., Humphries J. D., Craig S. E., Knight D. and Humphries M. J. (2012). Proteomic analysis of α4β1 integrin adhesion complexes reveals α-subunit-dependent protein recruitment. *Proteomics* 12, 2107-2114. 10.1002/pmic.201100487 22623428 PMC3472074

[JCS192054C7] Byron A., Askari J. A., Humphries J. D., Jacquemet G., Koper E. J., Warwood S., Choi C. K., Stroud M. J., Chen C. S., Knight D. et al. (2015). A proteomic approach reveals integrin activation state-dependent control of microtubule cortical targeting. *Nat. Commun.* 6, 6135 10.1038/ncomms7135 25609142 PMC4317495

[JCS192054C8] Carisey A., Tsang R., Greiner A. M., Nijenhuis N., Heath N., Nazgiewicz A., Kemkemer R., Derby B., Spatz J. and Ballestrem C. (2013). Vinculin regulates the recruitment and release of core focal adhesion proteins in a force-dependent manner. *Curr. Biol.* 23, 271-281. 10.1016/j.cub.2013.01.009 23375895 PMC3580286

[JCS192054C9] Chautard E., Ballut L., Thierry-Mieg N. and Ricard-Blum S. (2009). MatrixDB, a database focused on extracellular protein-protein and protein-carbohydrate interactions. *Bioinformatics* 25, 690-691. 10.1093/bioinformatics/btp025 19147664 PMC2647840

[JCS192054C10] Cole D. E. C. and Carpenter T. O. (1987). Bone fragility, craniosynostosis, ocular proptosis, hydrocephalus, and distinctive facial features: a newly recognized type of osteogenesis imperfecta. *J. Pediatr.* 110, 76-80. 10.1016/S0022-3476(87)80292-5 3794889

[JCS192054C11] Dong J.-M., Tay F. P.-L., Swa H. L.-F., Gunaratne J., Leung T., Burke B. and Manser E. (2016). Proximity biotinylation provides insight into the molecular composition of focal adhesions at the nanometer scale. *Sci. Signal.* 9, rs4 10.1126/scisignal.aaf3572 27303058

[JCS192054C12] Geiger B. and Yamada K. M. (2011). Molecular architecture and function of matrix adhesions. *Cold Spring Harb. Perspect. Biol.* 3, a005033 10.1101/cshperspect.a005033 21441590 PMC3101841

[JCS192054C13] Geiger T. and Zaidel-Bar R. (2012). Opening the floodgates: proteomics and the integrin adhesome. *Curr. Opin. Cell Biol.* 24, 562-568. 10.1016/j.ceb.2012.05.004 22728062

[JCS192054C14] Guo Z., Neilson L. J., Zhong H., Murray P. S., Zanivan S. and Zaidel-Bar R. (2014). E-cadherin interactome complexity and robustness resolved by quantitative proteomics. *Sci. Signal.* 7, rs7 10.1126/scisignal.2005473 25468996 PMC4972397

[JCS192054C15] Hoffmann J.-E., Fermin Y., Stricker R. L. O., Ickstadt K. and Zamir E. (2014). Symmetric exchange of multi-protein building blocks between stationary focal adhesions and the cytosol. *eLife* 3, e02257 10.7554/eLife.02257 24894463 PMC4040925

[JCS192054C16] Horton E. R., Byron A., Askari J. A., Ng D. H. J., Millon-Frémillon A., Robertson J., Koper E. J., Paul N. R., Warwood S., Knight D. et al. (2015). Definition of a consensus integrin adhesome and its dynamics during adhesion complex assembly and disassembly. *Nat. Cell Biol.* 17, 1577-1587. 10.1038/ncb3257 26479319 PMC4663675

[JCS192054C17] Horton E. R., Humphries J. D., Stutchbury B., Jacquemet G., Ballestrem C., Barry S. T. and Humphries M. J. (2016a). Modulation of FAK and Src adhesion signaling occurs independently of adhesion complex composition. *J. Cell Biol.* 212, 349-364. 10.1083/jcb.201508080 26833789 PMC4739608

[JCS192054C18] Horton E. R., Astudillo P., Humphries M. J. and Humphries J. D. (2016b). Mechanosensitivity of integrin adhesion complexes: role of the consensus adhesome. *Exp. Cell Res.* 343, 7-13. 10.1016/j.yexcr.2015.10.025 26515553

[JCS192054C19] Huang I.-H., Hsiao C.-T., Wu J.-C., Shen R.-F., Liu C.-Y., Wang Y.-K., Chen Y.-C., Huang C.-M., del Álamo J. C., Chang Z.-F. et al. (2014). GEF-H1 controls focal adhesion signaling that regulates mesenchymal stem cell lineage commitment. *J. Cell Sci.* 127, 4186-4200. 10.1242/jcs.150227 25107365 PMC4179489

[JCS192054C20] Humphries J. D., Byron A., Bass M. D., Craig S. E., Pinney J. W., Knight D. and Humphries M. J. (2009). Proteomic analysis of integrin-associated complexes identifies RCC2 as a dual regulator of Rac1 and Arf6. *Sci. Signal.* 2, ra51 10.1126/scisignal.2000396 19738201 PMC2857963

[JCS192054C21] Humphries J. D., Paul N. R., Humphries M. J. and Morgan M. R. (2015). Emerging properties of adhesion complexes: what are they and what do they do? *Trends Cell Biol.* 25, 388-397. 10.1016/j.tcb.2015.02.008 25824971

[JCS192054C22] Iwamoto D. V. and Calderwood D. A. (2015). Regulation of integrin-mediated adhesions. *Curr. Opin. Cell Biol.* 36, 41-47. 10.1016/j.ceb.2015.06.009 26189062 PMC4639423

[JCS192054C23] Jones M. C., Humphries J. D., Byron A., Millon-Frémillon A., Robertson J., Paul N. R., Ng D. H. J., Askari J. A. and Humphries M. J. (2015). Isolation of integrin-based adhesion complexes. *Curr. Protoc. Cell Biol.* 66, 9.8.1-9.8.15. 10.1002/0471143030.cb0908s66 PMC4402726 25727331

[JCS192054C24] Kanchanawong P., Shtengel G., Pasapera A. M., Ramko E. B., Davidson M. W., Hess H. F. and Waterman C. M. (2010). Nanoscale architecture of integrin-based cell adhesions. *Nature* 468, 580-584. 10.1038/nature09621 21107430 PMC3046339

[JCS192054C25] Köster J., Zamir E. and Rahmann S. (2012). Efficiently mining protein interaction dependencies from large text corpora. *Integr. Biol.* 4, 805-812. 10.1039/c2ib00126h 22706334

[JCS192054C26] Kuo J.-C., Han X., Hsiao C.-T., Yates J. R. III and Waterman C. M. (2011). Analysis of the myosin-II-responsive focal adhesion proteome reveals a role for β-Pix in negative regulation of focal adhesion maturation. *Nat. Cell Biol.* 13, 383-393. 10.1038/ncb2216 21423176 PMC3279191

[JCS192054C27] Kuo J.-C., Han X., Yates J. R. and Waterman C. M. (2012). Isolation of focal adhesion proteins for biochemical and proteomic analysis. *Methods Mol. Biol.* 757, 297-323. 10.1007/978-1-61779-166-6_19 21909920 PMC4158431

[JCS192054C28] Lavelin I., Wolfenson H., Patla I., Henis Y. I., Medalia O., Volberg T., Livne A., Kam Z. and Geiger B. (2013). Differential effect of actomyosin relaxation on the dynamic properties of focal adhesion proteins. *PLoS ONE* 8, e73549 10.1371/journal.pone.0073549 24039980 PMC3767655

[JCS192054C29] Lele T. P., Thodeti C. K., Pendse J. and Ingber D. E. (2008). Investigating complexity of protein–protein interactions in focal adhesions. *Biochem. Biophys. Res. Commun.* 369, 929-934. 10.1016/j.bbrc.2008.02.137 18331831 PMC2730744

[JCS192054C30] Morse E. M., Brahme N. N. and Calderwood D. A. (2014). Integrin cytoplasmic tail interactions. *Biochemistry* 53, 810-820. 10.1021/bi401596q 24467163 PMC3985435

[JCS192054C31] Ng D. H. J., Humphries J. D., Byron A., Millon-Frémillon A. and Humphries M. J. (2014). Microtubule-dependent modulation of adhesion complex composition. *PLoS ONE* 9, e115213 10.1371/journal.pone.0115213 25526367 PMC4272306

[JCS192054C32] Rauch F., Fahiminiya S., Majewski J., Carrot-Zhang J., Boudko S., Glorieux F., Mort J. S., Bächinger H.-P. and Moffatt P. (2015). Cole-Carpenter syndrome is caused by a heterozygous missense mutation in P4HB. *Am. J. Hum. Genet.* 96, 425-431. 10.1016/j.ajhg.2014.12.027 25683117 PMC4375435

[JCS192054C33] Rees J. S., Li X.-W., Perrett S., Lilley K. S. and Jackson A. P. (2015). Protein neighbors and proximity proteomics. *Mol. Cell. Proteomics* 14, 2848-2856. 10.1074/mcp.R115.052902 26355100 PMC4638030

[JCS192054C34] Robertson J., Jacquemet G., Byron A., Jones M. C., Warwood S., Selley J. N., Knight D., Humphries J. D. and Humphries M. J. (2015). Defining the phospho-adhesome through the phosphoproteomic analysis of integrin signalling. *Nat. Commun.* 6, 6265 10.1038/ncomms7265 25677187 PMC4338609

[JCS192054C35] Rolland T., Taşan M., Charloteaux B., Pevzner S. J., Zhong Q., Sahni N., Yi S., Lemmens I., Fontanillo C., Mosca R. et al. (2014). A proteome-scale map of the human interactome network. *Cell* 159, 1212-1226. 10.1016/j.cell.2014.10.050 25416956 PMC4266588

[JCS192054C36] Rossier O., Octeau V., Sibarita J.-B., Leduc C., Tessier B., Nair D., Gatterdam V., Destaing O., Albigès-Rizo C., Tampé R. et al. (2012). Integrins β1 and β3 exhibit distinct dynamic nanoscale organizations inside focal adhesions. *Nat. Cell Biol.* 14, 1057-1067. 10.1038/ncb2588 23023225

[JCS192054C37] Roux K. J., Kim D. I., Raida M. and Burke B. (2012). A promiscuous biotin ligase fusion protein identifies proximal and interacting proteins in mammalian cells. *J. Cell Biol.* 196, 801-810. 10.1083/jcb.201112098 22412018 PMC3308701

[JCS192054C38] Salmela M., Rappu P., Lilja J., Niskanen H., Taipalus E., Jokinen J. and Heino J. (2016). Tumor promoter PMA enhances kindlin-2 and decreases vimentin recruitment into cell adhesion sites. *Int. J. Biochem. Cell Biol.* 78, 22-30. 10.1016/j.biocel.2016.06.017 27373681

[JCS192054C39] Sarhan A. R., Patel T. R., Cowell A. R., Tomlinson M. G., Hellberg C., Heath J. K., Cunningham D. L., and Hotchin N. A. (2016). LAR protein tyrosine phosphatase regulates focal adhesions via CDK1. *J. Cell Sci.* 129, 2962-2971. 10.1242/jcs.191379 27352860

[JCS192054C40] Schiller H. B. and Fässler R. (2013). Mechanosensitivity and compositional dynamics of cell–matrix adhesions. *EMBO Rep.* 14, 509-519. 10.1038/embor.2013.49 23681438 PMC3674437

[JCS192054C41] Schiller H. B., Friedel C. C., Boulegue C. and Fässler R. (2011). Quantitative proteomics of the integrin adhesome show a myosin II-dependent recruitment of LIM domain proteins. *EMBO Rep.* 12, 259-266. 10.1038/embor.2011.5 21311561 PMC3059911

[JCS192054C42] Schiller H. B., Hermann M.-R., Polleux J., Vignaud T., Zanivan S., Friedel C. C., Sun Z., Raducanu A., Gottschalk K.-E., Théry M. et al. (2013). β1- and αv-class integrins cooperate to regulate myosin II during rigidity sensing of fibronectin-based microenvironments. *Nat. Cell Biol.* 15, 625-636. 10.1038/ncb2747 23708002

[JCS192054C43] Shtengel G., Galbraith J. A., Galbraith C. G., Lippincott-Schwartz J., Gillette J. M., Manley S., Sougrat R., Waterman C. M., Kanchanawong P., Davidson M. W. et al. (2009). Interferometric fluorescent super-resolution microscopy resolves 3D cellular ultrastructure. *Proc. Natl. Acad. Sci. USA* 106, 3125-3130. 10.1073/pnas.0813131106 19202073 PMC2637278

[JCS192054C44] Theodosiou M., Widmaier M., Böttcher R. T., Rognoni E., Veelders M., Bharadwaj M., Lambacher A., Austen K., Müller D. J., Zent R. et al. (2016). Kindlin-2 cooperates with talin to activate integrins and induces cell spreading by directly binding paxillin. *eLife* 5, e10130 10.7554/eLife.10130 26821125 PMC4749545

[JCS192054C45] Winograd-Katz S. E., Fässler R., Geiger B. and Legate K. R. (2014). The integrin adhesome: from genes and proteins to human disease. *Nat. Rev. Mol. Cell Biol.* 15, 273-288. 10.1038/nrm3769 24651544

[JCS192054C46] Wolfenson H., Lavelin I. and Geiger B. (2013). Dynamic regulation of the structure and functions of integrin adhesions. *Dev. Cell* 24, 447-458. 10.1016/j.devcel.2013.02.012 23484852 PMC3878073

[JCS192054C47] Wu J., Vallenius T., Ovaska K., Westermarck J., Mäkelä T. P. and Hautaniemi S. (2009). Integrated network analysis platform for protein-protein interactions. *Nat. Methods* 6, 75-77. 10.1038/nmeth.1282 19079255

[JCS192054C48] Yue J., Xie M., Gou X., Lee P., Schneider M. D. and Wu X. (2014). Microtubules regulate focal adhesion dynamics through MAP4K4. *Dev. Cell* 31, 572-585. 10.1016/j.devcel.2014.10.025 25490267 PMC4261153

[JCS192054C49] Zaidel-Bar R. and Geiger B. (2010). The switchable integrin adhesome. *J. Cell Sci.* 123, 1385-1388. 10.1242/jcs.066183 20410370 PMC2858016

[JCS192054C50] Zaidel-Bar R., Itzkovitz S., Ma'ayan A., Iyengar R. and Geiger B. (2007). Functional atlas of the integrin adhesome. *Nat. Cell Biol.* 9, 858-867. 10.1038/ncb0807-858 17671451 PMC2735470

[JCS192054C51] Zamir E. and Geiger B. (2001a). Components of cell-matrix adhesions. *J. Cell Sci.* 114, 3577-3579. 11707509 10.1242/jcs.114.20.3577

[JCS192054C52] Zamir E. and Geiger B. (2001b). Molecular complexity and dynamics of cell-matrix adhesions. *J. Cell Sci.* 114, 3583-3590. 11707510 10.1242/jcs.114.20.3583

